# Lung cancer symptoms and pulse oximetry in the prognostic assessment of patients with lung cancer

**DOI:** 10.1186/1471-2407-5-72

**Published:** 2005-07-06

**Authors:** Sandro J Martins, Nelson Ho, Sueli O Cavamura, Cecilia M Harada, Crystina A Yamamoto, Teresa Y Takagaki

**Affiliations:** 1 Pulmonary Division, University of São Paulo Medical School, Av. Dr. Arnaldo 455, 01246-903 São Paulo, Brazil; 2 Psychology Section, Hospital das Clínicas, University of São Paulo Medical School, Av. Dr. Arnaldo 455, 01246-903 São Paulo, Brazil; 3 Oncology Unit, Hospital Santa Izabel, Pca Almeida Couto 500, 40050-410 Salvador, Brazil

## Abstract

**Background:**

Medical oncologists continue to use performance status as a proxy for quality of life (QOL) measures, as completion of QOL instruments is perceived as time consuming, may measure aspects of QOL not affected by cancer therapy, and interpretation may be unclear. The pulse oximeter is widely used in clinical practice to predict cardiopulmonary morbidity after lung resection in cancer patients, but little is known on its role outside the surgical setting. We evaluated whether the Lung Cancer Symptom Scale and pulse oximetry may contribute to the evaluation of lung cancer patients who received standard anticancer therapy.

**Methods:**

We enrolled forty-one consecutive, newly diagnosed, patients with locally advanced or metastatic lung cancer in this study. We developed a survival model with the variables gender, age, histology, clinical stage, Karnofsky performance status, wasting, LCSS symptom scores, average symptom burden index, and pulse oximetry (SpO2).

**Results:**

Patient and observer-rated scores were correlated, except for the fatigue subscale. The median SpO2 was 95% (range: 86 to 98), was unrelated to symptom scores, and was weakly correlated with observer cough scores. In a multivariate survival model, SpO2 > 90% and patient scores on the LCSS appetite and fatigue subscales were independent predictors of survival.

**Conclusion:**

LCSS fatigue and appetite rating, and pulse oximetry should be studied further as prognostic factors in lung cancer patients.

## Background

The symptoms of lung cancer are a burden to patients, and a major detriment to their quality of life (QoL) and ability to function. Several different instruments have been developed to assess QoL of lung cancer patients, with recognized reliability and validity [1,2], yet their use is not widespread. Medical oncologists continue to use performance status as a proxy for QOL measures, as completion of QOL instruments is perceived as time consuming, may measure aspects of QOL not affected by cancer therapy, and interpretation may be unclear [3]. Whether formal QoL evaluation can improve patient care and disease outcome, perhaps through helping medical reasoning, by adding information to other well-known prognostic factors is a matter of active research.

The Lung Cancer Symptom Scale (LCSS) is a disease-and site-specific instrument developed in 1985 by Patricia Hollen and colleagues at the Memorial Sloan-Kettering Cancer Center, primarily to assess quality of life for individuals with lung cancer, both in its physical and functional aspects. It is focused on six clinically important lung cancer symptoms that are likely to affect patients' physical and functional status. Although it lacks detail in many QoL domains, such as social, spiritual, and psychological parameters, the LCSS has demonstrated high inter-rater reliability, feasibility, reliability, and content validity when evaluated against the Sickness Impact Profile (appetite), Profiles of Mood States (fatigue), American Thoracic Society Questionnaire (cough and dyspnea) and the McGill Pain Questionnaire, and their normative data in patients with non-small cell lung cancer (NSCLC) has been published [4-7].

Pulse oximetry is widely used to rapidly monitor arterial oxygen saturation (SpO2) [8]. It has many of the characteristics of an ideal monitoring technique: portability, non-invasiveness, ease of use (calibration is not required) and the capability for continuous on-line monitoring of SpO2. SpO2 measurement may be particularly useful in the care patients with advanced lung cancer, leading to early detection and appropriate management of hypoxemia, a condition likely to occur in this clinical setting [9].

In this study, we evaluate the prognostic value of baseline LCSS scores and pulse oximetry in a cohort of ambulatory patients, with advanced lung cancer, receiving standard medical therapy.

## Method

### Design

This was an observational cohort study. Consecutive outpatients with advanced or metastatic lung cancer who attended the pulmonology division of the Hospital das Clínicas of the University of São Paulo, Brazil, were invited to participate. All procedures were conducted in accordance with the ethical standards of the World Medical Association Declaration of Helsinki and Institutional board.

New patients admitted for lung cancer therapy were eligible if they presented with histological diagnosis of lung cancer, and locally advanced or metastatic disease extent. Exclusion criteria included past infection (last month) or fever (last week); use of supplementary oxygen; and potential causes for bad signal capture by pulse oximeter: black skin, nail abnormalities or pigmentation, anemia (hemoglobin < 10 g/dL), jaundice (bilirubin < 3 mg/dL), hypotension (systolic bp ≤ 90 mmHg), or tachycardia (> 100 bpm) were not included. All patients gave written signed informed consent before participating.

### Lung Cancer Symptom Scale

The LCSS comprises two scales, one for patients and other for health professionals as observer. The patient scale consists of nine items: six subscales related to major lung cancer symptoms (appetite, cough, dyspnea, fatigue, hemoptysis, and pain), all using 100 mm visual analogue measurements, and three summation items (activity status, symptomatic distress, and overall QoL). The observer scale is a five-point scale that measures the intensity of those symptoms (100 = none, 75 = mild, 50 = moderate, 25 = marked, and 0 = severe). The average symptom burden is an ancillary index, obtained as the mean of the six symptom scales scored separately by patients and the observer. At study admission, two psychologists explained all procedures and oriented patients to focus on the past day information about their symptom perception. A medical oncologist recorded symptom scores using the observer scale. In this study we used a Portuguese version of the LCSS developed for Brazil, through a forward and backward translation process [10].

### Pulse oximetry

The attending investigator at the initial medical examination recorded the mean of three consecutive finger pulse oximetry measures (Nonim Med Inc; Plymouth, USA), read at least 20 seconds apart with up to 2% variability, as the non-invasive estimate of SpO2.

### Medical therapy

Chemotherapy, radiotherapy, and supportive measures were administered on individual basis, and consisted of non-investigational therapies only. The attending physician was responsible for indicating the proper therapeutic modality according to patients' individual characteristics (comorbidities, clinical status, disease sites, and personal preferences). All chemotherapy regimens used were platinum-based.

### Data analysis

The following variables were selected for this analysis: gender, age (years), histology, clinical stage, Karnofsky performance status (KPS), weight loss (% usual body weight), symptom subscores (appetite, cough, dyspnea, fatigue, hemoptysis, and pain), average symptom burden index, and SpO2.

Due to non-normality constraints, bivariate association among symptom scores, Karnofsky performance status, and SpO2 were evaluated by nonparametric statistics (Spearman's rho). A Cox model was fit by step-wise procedure, as follows: a global test was performed to show the simple, unadjusted relationship between factors and survival, and seven possible explanatory variables were selected to model building due to its anticipated prognostic value (Wald chi square > 2.7); a stepwise model selection procedure was used and included explanatory variables that were significant at the 0.10 level; the likelihood ratio test was used to assess every improvement in model fitting. We used the SPSS package to perform statistical analysis (version 11.01, SPSS Inc, Chicago, IL).

## Results

From Oct 2000 to Apr 2001, forty-one consecutive eligible new patients with lung cancer were studied. Baseline demographic and clinical data are presented in Table 1. Our sample was largely comprised of aged, male, advanced lung cancer patients. Most patients (83%) had NSCLC and presented with poor performance status and weight loss. All patients received initial chemotherapy: in NSCLC, mitomycin C, vinblastine and cisplatin (61%) or carboplatin (22%); in small cell lung cancer, cisplatin and etoposide (17%). Nine patients (22%) received concurrent cisplatin radiotherapy (50.4 Gy) consolidation after four chemotherapy cycles. Median number of LCSS symptoms was 5 (range: 1 to 6); fatigue (95%) was the major presenting symptom, followed by pain (80%), dyspnea (78%), cough (73%), loss of appetite (68%), and hemoptysis (39%). All patients complied with LCSS administration.

### LCSS scores

To compare individual and health professional perceptions on symptom distress, the relationship between patient and observer LCSS scores are showed in Table 2. We expected significant correlation in all subscales since they measured the same distress dimension, but we found disagreement on fatigue evaluation.

Observer-derived average symptom burden index was not associated with age, histology, clinical stage, and KPS; except for hemoptysis (p = 0.11) and pain (p = 0.09), it was correlated with patient-derived subscales: appetite (rho = 0.33, p = 0.034), fatigue (rho = 0.34, p = 0.031), cough (rho = 0.54, p = 0.005), and dyspnea (rho = 0.46, p = 0.002). Patient-derived average symptom burden was associated with KPS (p = 0.001) and independent of age, histology, or clinical stage; it was also related to all but one obsever-derived symptom subscale (pain, p = 0.16): appetite (rho = 0.39, p = 0.012), fatigue (rho = 0.42, p = 0.006), cough (rho = 0.38, p = 0.014), dyspnea (rho = 0.52, p = 0.0001), and hemoptysis (rho = 0.39, p = 0.013).

### Pulse oximetry and LCSS scores

Median SpO2 was 95% (range: 86 to 98); eight patients (19%) had SpO2 below 90% but were not deemed candidates for domiciliary oxygen therapy. As presented in Table 3, the SpO2 measurement was unrelated to patient-derived symptom scores and exhibited only a weak correlation with observer-derived cough scores.

### Survival analysis

As of December 2003, thirty-eight patients (93%) had died and three were lost to follow up. The median survival was 42 weeks and 36% survived the first year. A multivariate survival model developed from seven possible explanatory variables is presented in Table 4. SpO2 > 90%, patient-derived scores on the appetite and fatigue subscales were found to be the only independent prognostic factors in this cohort. Clinical stage retained borderline prognostic value in the final model. Age (p = 0.92), gender (p = 0.79), and Karnofsky's performance status (p = 0.94) did not behave as independent prognostic factors. Global symptom scores failed to show relevant prognostic value on univariate tests and were not selected to the survival modeling.

## Discussion

Despite significant advances in cancer medicine, most physicians still deal with patients suffering from relentless, incurable illnesses, and their therapeutic decisions are often made based on subjective judgments about patients' QoL. Performance status scales, such as the KPS introduced in 1949, are widely accepted tools to evaluate physical functioning of cancer patients. Several recent studies suggest that a broad QoL evaluation using current well-structured instruments can provide more detailed prognostic information. This has been demonstrated for the Functional Living Index-Cancer [11,12], the European Organization for Research and Treatment of Cancer QLC43 form [2,13], and the Functional Assessment of Cancer Therapy – Lung questionnaire [14,15]. As shown here, the LCSS and pulse oximetry may be regarded as valuable tools for prognostic assessment in advanced lung cancer. SpO2 was independent of most LCSS data, and the combination of SpO2 and LCSS offered independent prognostic information on overall survival.

A SpO2 below 90% and variation of 10 mm in patient scores on appetite and fatigue were associated with an increased hazard of death. Measurement of pulse oximetry and completion of the LCSS form were fast and were not burdensome for the health care staff. The pulse oximeter is widely used in clinical practice to predict cardiopulmonary morbidity after lung resection in cancer patients [16], but little is known on its role outside the surgical setting.

The presence of anorexia and fatigue at initial evaluation has been associated with shortened overall survival. Loss of appetite is manifested by 75% of patients with advanced lung cancer at some point of their disease course [7], although in cancer centers its assessment eventually lead to therapeutic interventions in only 60% of the time [17]. Besides, its true prognostic value may be hidden or minimized in some studies given the fact that weight loss is usually the variable of interest. Recently, Hoang et al. [18] analyzed data from 1,436 patients with stage IV or IIIB NSCLC included in two randomized phase III Eastern Cooperative Oncology Group trials and found that loss of appetite conferred a hazard ratio for death of 1.62.

Fatigue also is a common symptom in patients with cancer, and is associated with both physical and psychological distress particularly patients with lung [19]. Using validated specific scales (Cancer Fatigue Scale and Fatigue Numerical Scale), fatigue that interferes with any daily activities may be detected in up to 59% of these patients [20]. Its independent negative impact on survival was reported in a retrospective study of 1,154 patients with lung carcinoma [21].

There are shortcomings on the use of SpO2 as a proxy measure of an estimate of SaO2 in patients with lung cancer. The presence of high carboxyhemoglobin values in heavy smokers could disturb the SpO2 reading by the pulse oximeter [22]. Many patients with lung cancer have some degree of chronic obstructive pulmonary disease (COPD), a condition in which SpO2 may not accurately represent arterial oxygen saturation [23]. Furthermore, the small sample size, heterogeneity of therapeutic approaches, and absence of full data on pulmonary comorbidity or carboxyhemoglobin values limit generalization of our model. Our four-factors model was fit from seven variables, from data on 41 patients and 38 events, and thus could not incorporate further prognostic information. In order to achieve reliable estimates of the three major functions (survival, probability density, and hazard) and their standard errors at each time interval by Cox regression the minimum recommended sample size should allow five to ten events per factor in the modeling set of variables [24,25].

In contrast to the patient's perspective, observers do not grade symptom distress only but also take into account the clinical setting related to that problem and discrepancies may rise. In the study presenting the conceptual model of the LCSS [26], fatigue was found to be the greatest significant predictor of symptomatic distress in NSCLC patients throughout therapy. We noticed a lack of correlation between patient and observer fatigue subscales, with a tendency for physicians to underrate this item. Although this may be due to misclassification errors on the categorical differences within the fatigue item by the observer, a bias towards an underestimation of symptom intensity by health professionals could not be ruled out. Discrepancies between observer and patient rated assessments have been reported for other symptom-based instrument, the Edmonton Symptom Assessment System [27,28].

To our knowledge, this is the first report suggesting the existence of a role for pulse oximetry in the assessment of newly diagnosed lung cancer patients receiving palliative anticancer therapy. Several issues still need to be clarified, including the pulse oximetry's performance under influence of smoking status or the coexistence COPD in lung cancer patients. Conceivably, its negative impact on prognosis could rely on the relationship of SpO2 with continued cigarette smoking, a condition associated to therapy failure and poor survival [29], or low SpO2 would reflect severity of underlying COPD. Our results add to the evidence that evaluation of lung cancer symptoms improve assessment of prognosis in the palliative setting, and support additional larger studies to refine and validate these findings so that they can be used in daily practice.

## Competing interests

The author(s) declare that they have no competing interests.

## Authors' contributions

SJM participated in the design of the study and performed the statistical analysis. NH participated in the patient care and carried out the pulse oximetry measurements. CAY, SOC and CMH participated in the design of the study, and carried out QOL measurements and their analysis. TYT conceived of the study, and participated in its design and coordination. All authors read and approved the final manuscript.

## Pre-publication history

The pre-publication history for this paper can be accessed here:



## Figures and Tables

**Table 1:** Patient characteristics

Median age (range), in years	63	(34 to 80)
		
Gender		
Male	32	(78%)
Female	9	(22%)
		
Karnofsky performance status		
60 %	3	(7%)
70 %	22	(54%)
80%	11	(27%)
90%	5	(12%)
		
Clinical Stage		
IIIA	7	(17%)
IIIB	12	(29%)
IV	22	(54%)
		
Histology		
Adenocarcinoma	16	(39%)
Squamous cell carcinoma	9	(22%)
Poorly differentiated carcinoma	6	(15%)
Large cell carcinoma	3	(7%)
Small cell carcinoma	7	(17%)
		
Metastatic sites		
Bone	8	(19%)
Addrenal	6	(15%)
Skin	4	(10%)
Lung	3	(7%)
Brain	2	(5%)
Liver	1	(2%)
		
Wasting	22	(54%)

**Table 2:** Correlation between patient and observer scores on symptom subscales

	Patient scale (mm) *	Observer scale (point) *	Spearman's rho	p-value†
Appetite	31 (19–43)	75 (50–100)	-0.52	0.0001
Fatigue	56 (46–65)	50 (0–100)	-0.21	N.S.
Cough	29 (19–39)	75 (25–100)	-0.71	0.0001
Dyspnea	38 (27–49)	75 (0–100)	-0.51	0.001
Pain	44 (32–57)	75 (25–100)	-0.36	0.02
Hemoptysis	13 (0–22)	100 (50–100)	-0.50	0.001
Global symptom score	35 (28–42)	69 (42–100)	-0.57	0.0001

* Median values and range.

† N.S.: Not significant at the 0.05 level (two-tailed)

**Table 3:** Correlation between pulse oximetry and LCSS subscales

	Patient scale		Observer scale	
	
	Spearman's rho	p-value *	Spearman's rho	p-value
Appetite	0.02	N.S	-0.20	N.S.
Fatigue	-0.23	N.S	-0.19	N.S.
Cough	-0.16	N.S	-0.33	0.03
Dyspnea	0.07	N.S	-0.26	N.S.
Pain	-0.13	N.S	0.02	N.S.
Hemoptysis	-0.85	N.S	-0.20	N.S.
Global symptom score	-0.23	N.S.	-0.31	0.049

* N.S.: Not significant at the 0.05 level (two-tailed)

**Table 4:** Survival analysis by Cox regression

	β	S.E.	OR	CI 95%	p-value
Pulse oximetry (SpO2 < 95%)	1.39	0.63	4.02	1.16–13.85	0.022
Clinical stage (III vs. IV)	0.78	0.43	2.18	0.93–5.06	0.070
Appetite (10 mm)	0.13	0.06	1.14	1.01–1.30	0.041
Fatigue (10 mm)	0.21	0.09	1.23	1.03–1.47	0.019

Overall model: Chi-square = 10.21, p = 0.037

Variables not in the equation: age, gender, and Karnofsky performance status
